# An unusual case of uterine cotyledonoid dissecting leiomyoma with adenomyosis

**DOI:** 10.1186/s13000-016-0523-1

**Published:** 2016-08-04

**Authors:** Ai Shimizu, Hoshihito Tanaka, Sari Iwasaki, Yukio Wakui, Hitoshi Ikeda, Akira Suzuki

**Affiliations:** 1 Department of Pathology, KKR Sapporo Medical Center, 6-3–40, Ichijyo, Hiragishi, Toyohira-ku, Sapporo, Hokkaido 062-0931 Japan; 2 Department of Gynecology, KKR Sapporo Medical Center, 6-3–40, Ichijyo, Hiragishi, Toyohira-ku, Sapporo, Hokkaido 062-0931 Japan; 3 Department of Physical Therapy, Faculty of Human Science, Hokkaido Bunkyo University, 196-1, 5-chome, Koganechuo, Eniwa 061-1449 Japan

**Keywords:** Leiomyoma of the uterus, Cotyledonoid dissecting leiomyoma, Adenomyosis, Adenomyoma

## Abstract

**Background:**

Cotyledonoid dissecting leiomyoma is a rare variant of uterine smooth muscle tumor with an unusual growth pattern that shows intramural dissection within uterine myometrium and often a placenta-like appearance in its extrauterine components.

**Case presentation:**

We present a unique case of cotyledonoid dissecting leiomyoma with adenomyosis. A 40-year-old Japanese female presented with prolonged menorrhagia and severe anemia. She had a pelvic mass followed-up for 6 years with a diagnosis of leiomyoma. However, increase in tumor size and cystic changes with hemorrhage were found by magnetic resonance imaging, and total abdominal hysterectomy with bilateral salpingectomy was performed. Macroscopically, the placenta-like exophytic mass protruding from the posterior uterine wall was composed of multiple nodules containing numerous hemorrhagic cysts. The mass showed continuity as a white multinodular dissecting mass infiltrating the posterolateral myometrium. Microscopically, both extra–and intrauterine portions of the mass were composed of nodules that contained swirled neoplastic smooth muscle cells with marked hyalinized degeneration, as observed in cotyledonoid dissecting leiomyomas of conventional type. In addition, numerous non–neoplastic glands of endometrial type surrounded by abundant endometrium–like stromal cells and non–neoplastic smooth muscle cells were found in the tumor, suggesting that it involved a part of concomitant adenomyosis originating from the nontumoral myometrium.

**Conclusions:**

Thus far, over 30 cases of cotyledonoid dissecting leiomyoma have been reported, none of which have described the presence of adenomyosis within the tumor. The present case suggested that cotyledonoid dissecting leiomyoma might have a unique clinical presentation involving concomitant uterine adenomyosis. It is critical for pathologists, gynecologists, and radiologists to be cognizant of cotyledonoid dissecting leiomyoma variants for timely and appropriate diagnosis and treatment.

## Background

Uterine leiomyomas are the most common type of uterine tumor [1], with its variants accounting for approximately 10 % of cases [1]. Cotyledonoid dissecting leiomyoma is an extremely rare variant of uterine leiomyoma with an unusual pattern that is characterized by intramural dissection within the uterine corpus and often a placenta–like macroscopic appearance of its extrauterine component [2]. David et al. originally reported this variant as grape–like leiomyoma in 1975 [3], which was later named as cotyledonoid dissecting leiomyoma in a series of four cases by Roth et al. [2]. Thereafter, over 30 cases have been reported thus far; all followed a clinically benign course [4–6]. Although several cases indicated coexisting focal endometriosis, endosalpingiosis, and adenomyomatous components [7–9], none showed obvious and abundant nontumoral components within the tumor. Here we present a unique case of cotyledonoid dissecting leiomyoma involving numerous non-neoplastic endometrial glands with endometrial stromal cells, indicating adenomyosis. To the best of our knowledge, this is the first description of cotyledonoid dissecting leiomyoma with ademomyosis.

## Case presentation

**Fig. 1:**
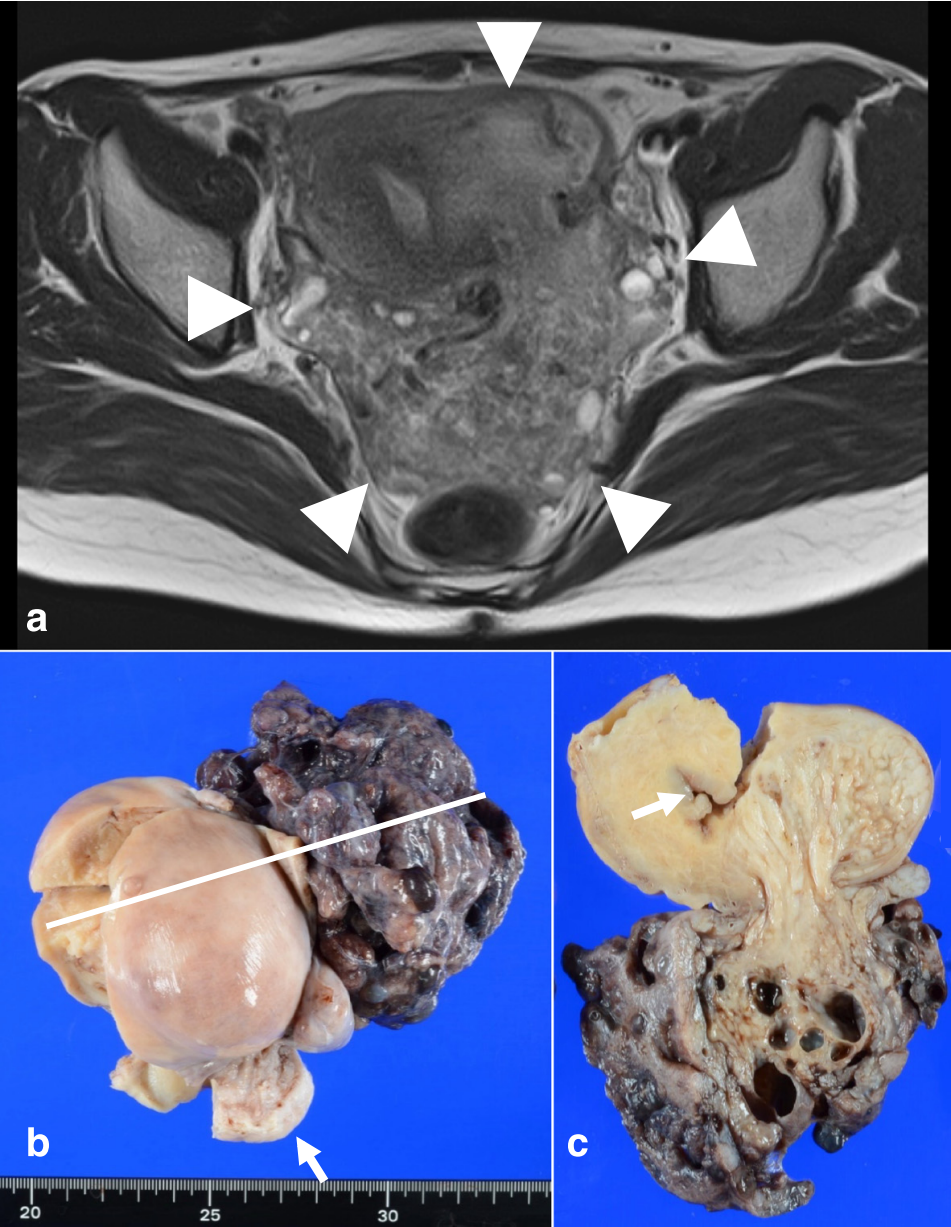
Magnetic resonance imaging and gross appearance of the tumor. **a** Axial T2-weighted image shows a pelvic mass, measuring 10 × 9 × 7 cm, located in the left posterolateral myometrial wall, with extrauterine extension to the pelvic cavity (arrowhead). **b** Macroscopically, a large exophytic mass protruding from the posterior uterine wall into the pelvic cavity is observed. The mass contains multiple deep red multiple nodules, giving it a placenta-like appearance. Uterine cervix is not affected (arrow). White line demonstrates the cut line shown on Fig. 1**c**. **c** The extrauterine mass contains numerous hemorrhagic cysts. The extrauterine mass shows continuity as a white indistinct multinodular mass in the myometrium. Endometrium is preserved (arrow)

A 40-year-old Japanese female with gravidity 2 and parity 2 presented with prolonged menorrhagia and severe anemia. The patient had a history of cervical carcinoma in situ and underwent cervical conization 10 years ago. During postoperative follow-up, carcinoma did not recur, but menorrhagia and anemia persisted. Six years before admission, a uterine leiomyoma 3.5 cm in diameter was confirmed by transvaginal ultrasound examination; the tumor increased in size to 5 cm in diameter during evaluation 2 years before admission. During the last year, an extrauterine mass 5 cm in diameter protruding from the posterior uterine wall was discovered. For further assessment and treatment, the patient was admitted to the gynecology department at our hospital. Magnetic resonance imaging showed a large pelvic mass measuring 10 × 9 × 7 cm (Fig. 1a) containing hemorrhagic cysts. The mass was located in the left posterolateral myometrial wall with an extrauterine extension. Total abdominal hysterectomy with bilateral salpingectomy was performed for suspicion of uterine sarcoma or degenerated leiomyoma.

### Pathological findings

**Fig. 2:**
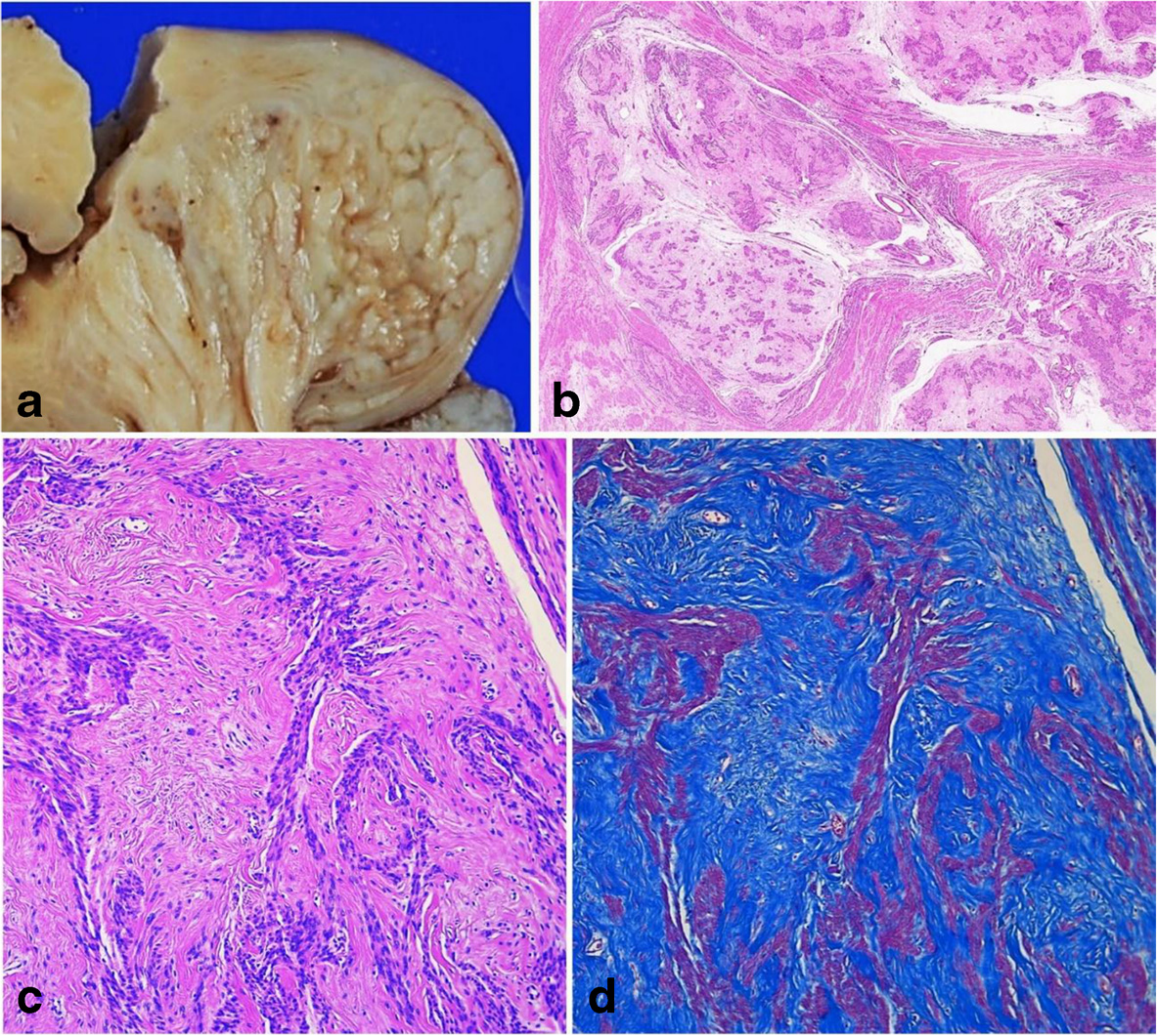
Macroscopic and histopathological features of the tumor. **a** The intrauterine mass contains multiple nodules. **b** Both extra- and intrauterine components parts consist of numerous ill-defined tumorous nodules, which dissect myometrium in the intrauterine part. All nodules show sparsely distributed swirls of benign smooth muscle cells, with marked hyalinized degeneration, and hydropic change (hematoxylin-eosin staining; object lens magnification, ×1.25). **c** The neoplastic smooth muscle cells are uniform, with spindle shaped nuclei and eosinophilic cytoplasm (hematoxylin-eosin staining; object lens magnification, ×10). **d** Masson trichrome staining reveals abundant hyalinized collagen fibers admixed with neoplastic myocyte fascicles in all nodules (object lens magnification, ×10)

Macroscopically, the placenta-like extrauterine mass measuring 10 × 9 × 9 cm was protruding from the posterior uterine wall (Fig. 1b). This mass consisted of multiple nodules with a deep red color giving it a placenta-like appearance and contained numerous hemorrhagic cysts measuring up to 2 cm in diameter (Fig. 1c). The exophytic mass showed continuity as a white dissecting multinodular mass measuring 4 × 3 × 3 cm in the left lateral myometrial wall (Fig. 1c and 2a). The rest of the myometrium was diffusely thickened but did not contain any nodular lesions.

**Fig. 3:**
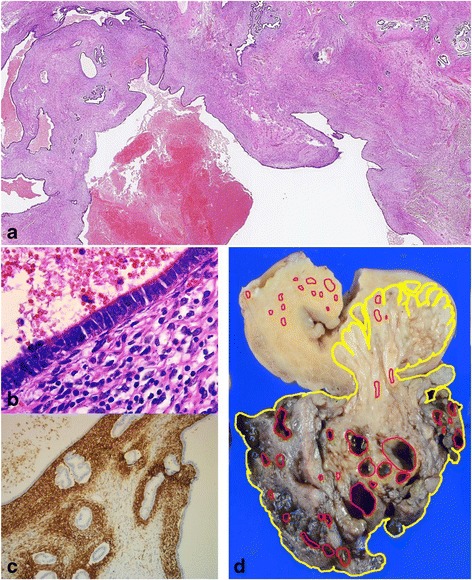
Histopathological and immunohistochemical findings of cystic lesions of the tumor. **a** Cystic and dilated endometrial glands are present in the extrauterine component, with degenerated blood inside (hematoxylin-eosin staining; object lens magnification, ×1.25). **b** The cyst is lined by one layer of columnar cells that resemble endometrial epithelium without cellular atypia and is surrounded by stromal cells (hematoxylin-eosin staining; object lens magnification, ×40). **c** The stromal cells are positive for CD10, an endometrial stromal marker (immunohistochemistry; object lens magnification, ×10). **d** Lesions of cotyledonoid dissecting leiomyoma (yellow) and adenomyosis (red) are mapped in the gross specimen shown in Fig. 1**c**, based on microscopic examination. Adenomyosis lesions are located not only in the extrauterine component but also in the intrauterine tumor lesion and thickened tumor-free myometrium

**Fig. 4:**
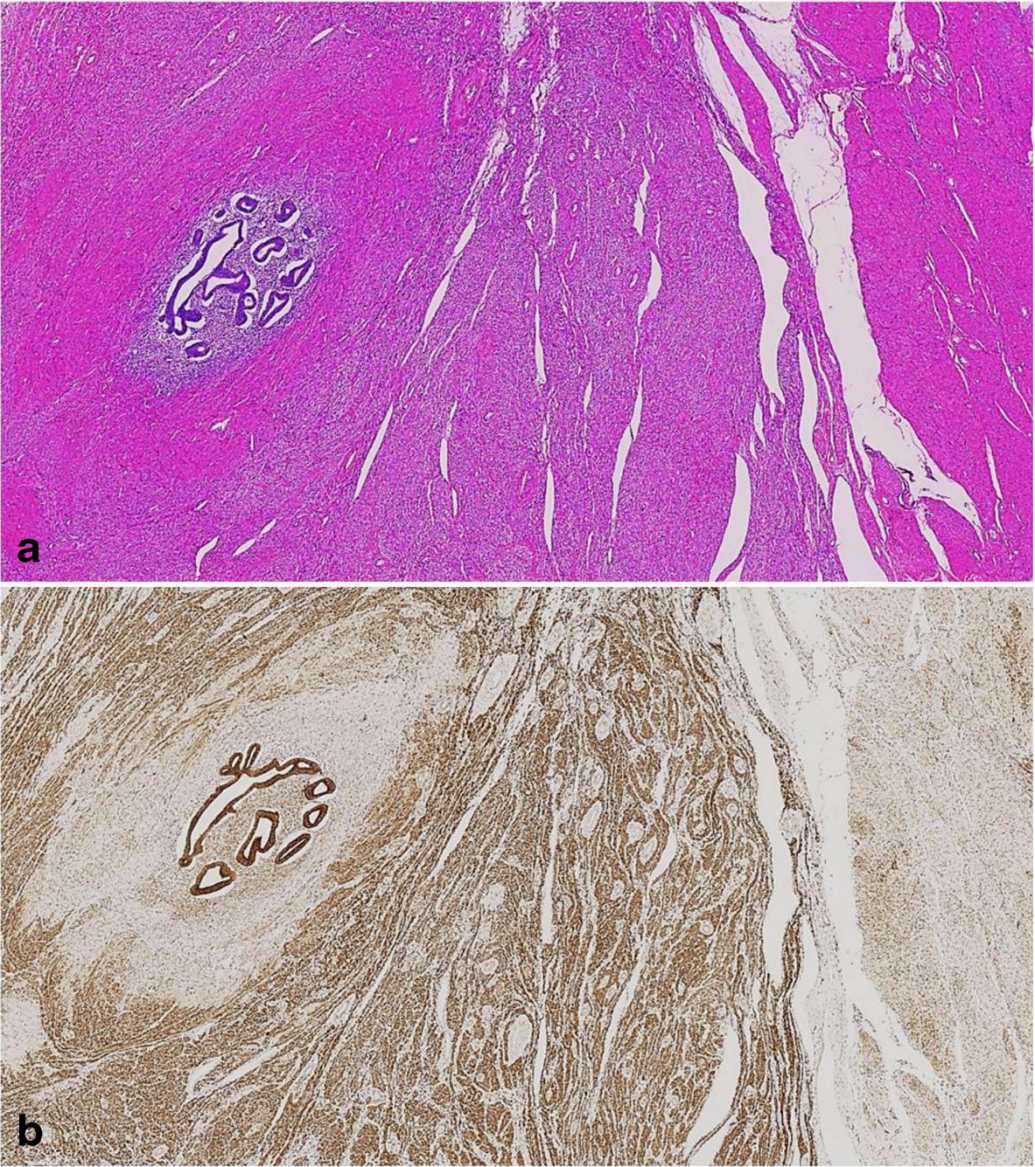
Histopathological findings and immunohistochemical staining of Bcl-2. **a** Left three quarters of the specimen show the tumor containing endometrial glands and stromal cells surrounded by several layers of smooth muscle cells. Non-neoplastic myometrium is on the right quarter of the specimen (hematoxylin-eosin staining; object lens magnification, ×2.5). **b** Negative to slightly positive Bcl-2 immunostaining is observed in the surrounding layers of smooth muscle cells and tumor-free myometrium (right one quarter of the specimen), whereas Bcl-2 protein expression in the cytoplasm of tumoral smooth muscle cells is abundant (immunohistochemistry; object lens magnification, ×2.5)

Histologically, both extra–and intrauterine components of the mass consisted of numerous ill–defined nodules, with nodules of the intrauterine component dissecting the myometrium (Fig. 2b). All nodules exhibited sparsely distributed swirls of benign smooth muscle cells, with marked hyalinized degeneration and hydropic change (Fig. 2b). The myocytes were uniform with slightly enlarged and bland spindle–shaped nuclei and eosinophilic cytoplasm (Fig. 2c). Mitotic figures, significant nuclear atypia, and coagulative tumor necrosis suggesting malignancy were not identified. Abundant collagen fibers were found among myocyte fascicles (Fig. 2d). Numerous congested and dilated vessels and edematous connective tissue between nodules were also found prominently in the extrauterine component of the mass. These macroscopic and histologic features of the tumor were similar to those of previously reported cases of cotyledonoid dissecting leiomyoma of the uterus [2–4]. In addition, numerous glands composed of columnar epithelium resembling endometrial gland epithelium were surrounded by stromal cells with features of endometrial stroma in both the extra-and intrauterine tumor components. These glands were markedly dilated in the extrauterine component and were concordant with gross hemorrhagic cysts (Fig. 3a and d). The endometrial gland-like columnar epithelial cells were nonciliated without cellular atypia (Fig. 3b), whereas the stromal cells were diffusely positive for CD10 (monoclonal, #56C6; Leica Biosystems, Newcastle, UK), an endometrial stromal marker, by immunohistochemistry (Fig. 3c); these findings were similar to the endometrial component of adenomyosis. Interestingly, some of the glands were enclosed by several layers of smooth muscle cells with myometrial features (Fig. 4a). These smooth muscle cells were negative to slightly positive for Bcl-2 (monoclonal, #124; DakoCytomation, Glostrup, Denmark) by immunohistochemistry, similar to that observed in tumor-free myometrium, whereas Bcl-2 expression was abundant in leiomyoma cells (Fig. 4b). In addition, ordinary adenomyosis was found in the thickened and tumor-free myometrium (Fig. 3d). All together, these findings suggested that the glands found in the tumor mass were part of the adenomyosis that were invaded by tumor growth. Endometriosis was not found in any other parts of the resected uterus or the fallopian tubes. The patient showed no recurrence at 3 months of postoperative follow-up.

## Discussion

In the present case, the clinical manifestation, gross appearance, and histological findings were in good agreement with typical cotyledonoid dissecting leiomyoma. Leiomyomas of the uterus are the most common uterine tumor and usually affect women in their fourth and fifth decades [1]. Most uterine leiomyomas are conventional types, whereas variant forms comprise approximately 10 % of all leiomyoma cases [1]. Cotyledonoid dissecting leiomyoma is an extremely rare variant of uterine leiomyoma with an unusual growth pattern characterized by an extrauterine mass that resembles a placenta and histologically shows an intramural dissecting pattern in the uterus [2, 3]. Saeed et al. reviewed 20 cases of cotyledonoid dissecting leiomyoma [4]. The ages of patients ranged from 23 to 65 years (mean, 40.3 years), which were less than those of patients with conventional leiomyomas. The most common clinical presentation of this variant is a pelvic mass and is followed by abnormal uterine bleeding. The size of these tumors ranged from 10 to 41 cm (mean, 17.7 cm). Thus far, over 30 cases have been reported in the literature [4, 5]. Histologically, the neoplastic smooth muscle cells form disorganized fascicles in cotyledonoid dissecting leiomyomas, in contrast to the organized pattern observed in conventional leiomyomas [6]. In this case, both the intrauterine and extrauterine part of the tumor was composed of the same nodular masses of disorganized smooth muscle cells and collagen fibers as those of previous reports (Fig. 2c and d). All cases reported to date were clinically benign, and only one case of recurrence after partial tumorectomy was reported [10]. Cases of epithelioid cotyledonoid dissecting leiomyoma variant [5] and cotyledonoid dissecting leiomyoma with intravenous leiomyomatosis [7] were also recently reported. However, neither an epithelioid pattern nor intravascular tumor components were found in our case.

Interestingly, several reports alluded to cotyledonoid dissecting leiomyomas with non-neoplastic cystic lesions (Table 1). Conventional uterine leiomyoma does not usually contain cystic lesions, whereas one case of intravenous leiomyomatosis of the uterus with an endometrial component was previously reported [11]. In addition, Fukunaga et al. described a cotyledonoid dissecting leiomyoma with limited foci of endometriosis in 1998 [8]; the foci were located in the shallow portion of the extrauterine components and the left ovary and were considered as endometriosis. Conversely, Driss et al. reported a cotyledonoid dissecting leiomyoma case associated with endosalpingiosis in 2009 [9]. The lesion was within the exophytic component with small cysts of 1–2 mm in diameter. These glands and cysts were lined by ciliated tubal type epithelium and surrounded by scanty, loose fibrous tissue. Immunohistochemistry revealed that the columnar epithelium was positive for cytokeratin 7, but the stromal cells were not characterized in detail. In a series of six cases, Jordan et al. described one tumor demonstrating cystic spaces within the extrauterine component [7]. They reported the presence of tubo-endometrial glands without accompanying stroma; thus, that finding was interpreted as an unusual adenoleiomyomatous element. The cotyledonoid dissecting leiomyoma in our patient presented with the greatest extent of non-neoplastic cystic lesions compared with the three previous cases. However, all these cases, including ours, suggest that cotyledonoid dissecting leiomyoma might have a unique clinical presentation involving parts of benign non-neoplastic cystic lesions.

**Table 1:** Summary of all reported cases of cotyledonoid dissecting leiomyoma with non-neoplastic cystic lesions

*Case*	*Age*	*Presentation*	*Tumor location*	*Size* (*cm*)	*Other components*	*Background*	*References*
*1*	35	Abdominal pain and mass	Lateral wall	18	Endometriosis	Endometriosis in left ovary	[9]
*2*	47	Abdominal mass	Posterior wall, broad ligaments, pelvic cavity	25	Endosalpingiosis	NA	[10]
*3* ^a^	NA	NA	NA	NA	Adenoleiomyomatous component	NA	[8]
*4*	40	Menorrhagia and severe anemia	Posterolateral wall, pelvic cavity	14	Adenomyosis	Adenomyosis	Present case

*NA* not available

^a^The case was one of the six cotyledonoid dissecting leiomyoma cases in reference [7]

We suppose the endometrial component in this tumor may be a part of adenomyosis, not endometriosis. It is because localization of the endometrial component is not superficial and endometriosis is not found in any part of the pelvic cavity. However, adenomyosis is distributed both in the tumor and tumor-free myometrium (Fig. 3d). Furthermore, some of these endometrial elements within the tumor are enclosed by non-neoplastic myometrial smooth muscle that is slightly immunopositive for Bcl-2 (Fig. 4a and b). Bcl-2 protein is known to be an apoptosis-inhibiting gene product, and it is also known to prevent apoptotic cell death in a variety of cells. Matsuo et al. reported that Bcl-2 immunopositivity was prominent in uterine leiomyoma cells and was scarcely present in normal myometrial smooth muscle cells [12]. They supposed that Bcl-2 protein associated with progesterone is responsible for the growth of leiomyomas by preventing apoptotic cell death [13].

On the other hand, adenomyoma should be an important differential diagnosis in the extrauterine part of the tumor. Adenomyoma is one of the mixed epithelial and mesenchymal tumors of the uterine corpus and is described as a well-circumscribed tumor composed of endometrial glands and endometrial-type stroma surrounded by abundant smooth muscle component [1]. We cannot definitely distinguish adenomyoma with involved adenomyosis in a cotyledonoid dissecting leiomyoma, because each histological component is very similar and also because of degenerative changes in the extrauterine part caused by menstrual bleeding followed by inflammation. Typical adenomyomas are intramural, firm, and smooth-surfaced tumors [14]. They show gray-white surfaces and are well demarcated from the myometrium on cut sections, and their smooth muscle component shows hypertrophy but does not make nodular proliferation with collagen fibers [15]. However, we can also find a few previous case reports of adenomyoma with prominent cystic change extended to the pelvic cavity [16, 17]. Since histological analysis of these tumors is not provided in detail, we cannot distinguish them from cotyledonoid dissecting leiomyoma with adenomyosis. Accumulation of similar cases will help us understand the nature and the difference between adenomyoma and cotyledonoid dissecting leiomyoma with adenomyosis.

The mechanism of tumor development in this patient is interesting. In this case, leiomyoma was first found in the uterine wall by transvaginal ultrasonography and was later noted to extend into the pelvic cavity. It is possible that during tumor growth from the myometrium, endometrial glands and stromal cells might have been captured by the intramural component of tumor during its dissection through the surrounding adenomyosis, to be located together with the extrauterine component. In this case, glandular components and stromal cells were also recognized in the intramural component, supporting this possibility. The cystic expansion of endometrial glands outside the uterus might be correlated with loose connective tissue with congestion and hydropic change.

The cotyledonoid dissecting leiomyoma in our case included a large number of non-neoplastic cystic lesions compared with previously reported three cases. This case indicated that cotyledonoid dissecting leiomyoma could occur with concomitant adenomyosis, depending on the pattern of tumor growth. These four cases, including ours, suggested that cotyledonoid dissecting leiomyoma might have a unique presentation with benign non-neoplastic cystic lesions, such as endometriosis, endosalpingiosis, adenoleiomyomatous component, and adenomyosis.

## Conclusions

The case presented herein is the first documented cotyledonoid dissecting leiomyoma with diffuse adenomyosis and reveals a unique variant of cotyledonoid dissecting leiomyoma involving benign cystic lesions. Accumulation of similar case reports is expected and important. While cotyledonoid dissecting leiomyoma has alarming gross appearance with hemorrhagic cysts of adenomyosis or other benign cystic lesions within the tumor mass, it has a completely benign clinical course. Gynecologists, radiologists, and pathologists should be cognizant of the variance and nature of cotyledonoid dissecting leiomyomas to avoid misdiagnosis and overtreatment.

## Abbreviations

Bcl-2, B-cell lymphoma 2; CD10, Cluster of differentiation 10
